# Efficacy and tolerability of lurasidone in schizophrenia: A systematic review and meta-analysis of short-term, randomized, placebo controlled trials

**DOI:** 10.1192/j.eurpsy.2021.424

**Published:** 2021-08-13

**Authors:** K. Hagi, T. Nosaka, J. Kane, C. Correll

**Affiliations:** 1 Medical Affairs, Sumitomo Dainippon Pharma Co., Ltd., Tokyo, Japan; 2 Psychiatry Research, The Zucker Hillside Hospital, Glen Oaks, United States of America; 3 Department Of Child And Adolescent Psychiatry, Psychosomatic Medicine And Psychotherapy, Charité University Medical Center, Berlin, Charite Berlin, Padua, Germany

**Keywords:** lurasidone, meta-analysis, RCT, schizophrénia

## Abstract

**Introduction:**

Lurasidone is an atypical antipsychotic approved for the treatment of patients with schizophrenia. We report on a meta-analysis focusing on both the efficacy and safety/tolerability of lurasidone in the treatment of patients with schizophrenia.

**Objectives:**

To obtain pooled estimates from placebo-controlled clinical trials on the efficacy and safety/tolerability of lurasidone in schizophrenia.

**Methods:**

We selected acute, randomized placebo-controlled trials of lurasidone for schizophrenia. Primary outcome for efficacy was the Positive and Negative Syndrome Scale (PANSS) change and for “acceptability” was all-cause discontinuation. Secondary outcomes included specific adverse events, body weight change, ≥7% weight gain, and glucose and lipid parameter change.

**Results:**

Across 10 RCTs (n=3,963, age=40.5±2.3 years, males=64.7 %, trial duration=6.0 weeks), lurasidone outperformed placebo regarding the PANSS total score (N=10, n=3,354, SMD=-0.34, 95% CI: -0.47−-0.21, p<0.001). Stratifying the analysis by dose, lurasidone significantly outperformed placebo at doses 40-160 mg/day. Lurasidone was associated with significantly lower all-cause discontinuation than placebo (N=10, n=3,410, RR=0.87, 95% CI: 0.78−0.97, p=0.014). Lurasidone had significantly higher body weight change compared with placebo (N=10, n=3,359, SMD=0.17, 95% CI: 0.09−0.24, p<0.001), but without significant differences regarding ≥7% body weight gain (N=9, n=3,186, p=0.112). Lurasidone did not differ from placebo in total cholesterol (N=10, n=3,140, p=0.439), LDL-cholesterol (N=7, n=2,414, p=0.849), triglycerides (N=10, n=3,140, p=0.238), and fasting glucose change (N=10, n=3,112, p=0.633).

**Conclusions:**

In short-term trials, lurasidone was efficacious, acceptable and safe, having minimal effect on body weight gain and glucose and lipid metabolism.

**Disclosure:**

K. Hagi is a full time employee of Sumitomo Dainippon Phrma Co., Ltd.

